# Supplementary material for: Effects of Load Carriage on Postural Control and Spatiotemporal Gait Parameters during Level and Uphill Walking
Source: Sensors (Basel). 2023 Jan 5;23(2):609. doi: 10.3390/s23020609 (PMC9863443; doi:10.3390/s23020609)
Supplement: Supplementary file 1 [file sensors-23-00609-s001.zip › sensors-1867682-supplementary.pdf]

**Supplementary Materials**

Six subjects were tested twice, with and without contact of the IMU and EMG electrodes with the front/backpack, to investigate the effect of possible interferences on the signals. Contact between the IMU and EMG electrodes with the front/backpack was prevented by placing specially shaped hard cardboard around both the IMU and the EMG electrodes.

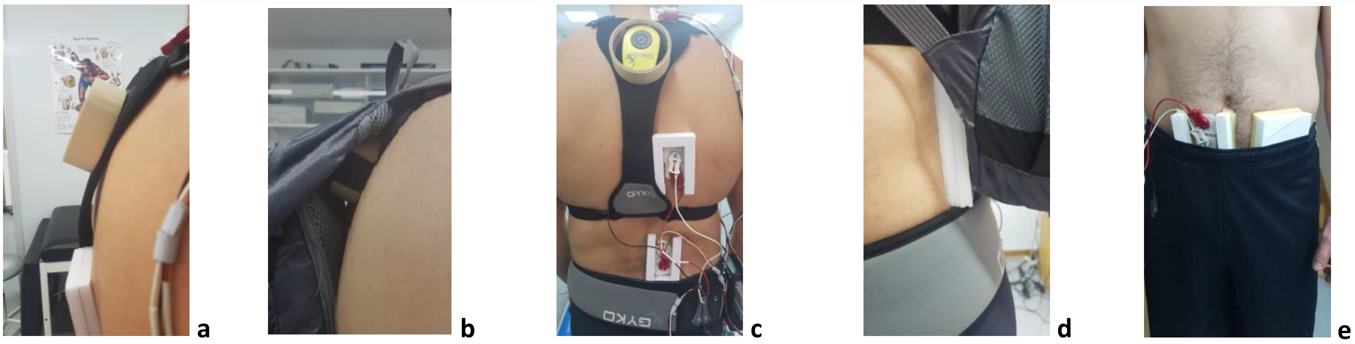


**Figure S1.** Locations of specially shaped hard cardboards placed around both the IMU (a-b) and the EMG electrodes located on the thoracic and lumbar erector spinae (c-d) as well as on rectus abdominis (e) to prevent contact with the carried load.

The potential interference of (i) the back pack with the IMU and the EMG electrodes placed on the thoracic and lumbar erector spinae during BL walking and (ii) the front pack with the EMG electrodes placed on the rectus abdominis during FL walking, were examined at all slopes under investigation. The statistical differences between IMU/EMG data acquired with and without contact of the sensors with the front or the backpack was investigated using paired t-test.

Non-contact of the IMU and the EMG electrodes with the front back or the backpack did not significantly affect the recording of the signals. Furthermore, both trunk sway and muscle activity demonstrated the same pattern of increase as the treadmill's slope increased with participants walking with both contact and non-contact of the IMU and EMG electrodes with the front back or the backpack, respectively.

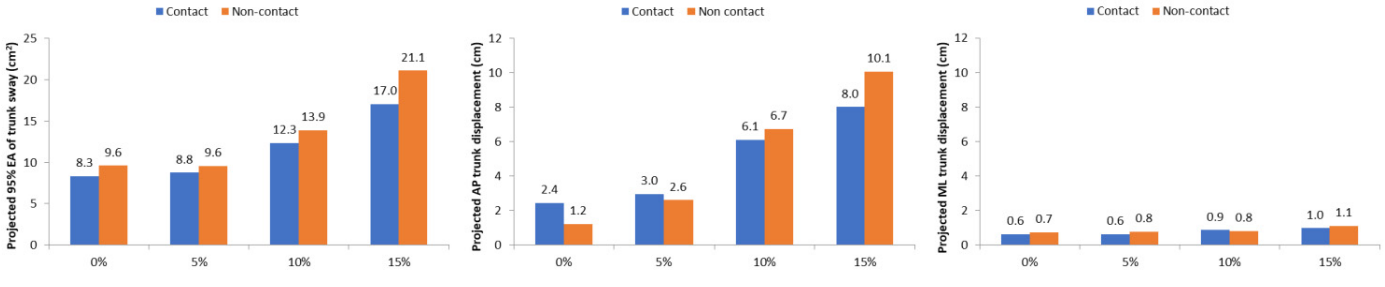


a b c

**Figure S2.** Graphs depicting trunk sway-based data such as the 95% EA (a), and the projected AP (b) and ML trunk displacement (c) acquired with and without contact of the IMU sensor with the backpack during level and uphill BL walking.

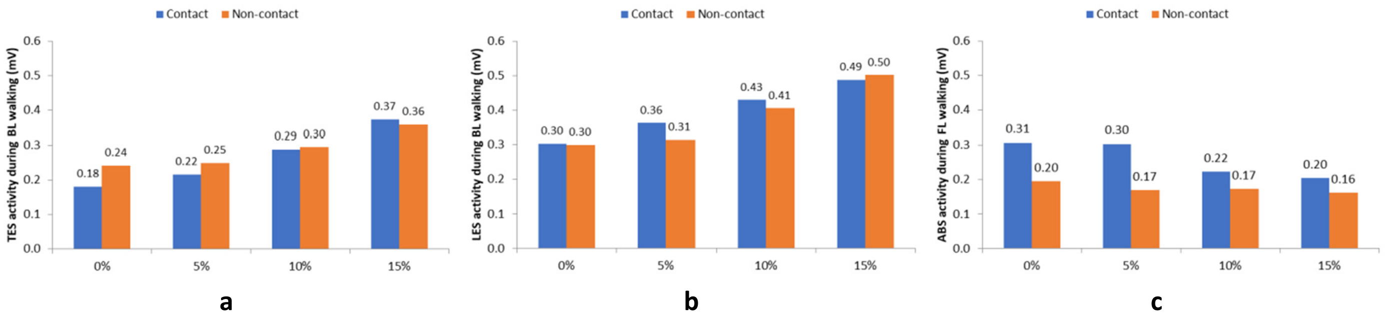


**Figure S3.** Graphs depicting the TES **(a)** and LES **(b)** the EMG activity recorded during level and uphill BL walking as well as the RAB **(c)** activity recorded during level and uphill FL walking.
